# Identification of age-group reservoirs for persistent vaccine-type pneumococcal carriage in rural Gambia

**DOI:** 10.1186/s41479-025-00176-w

**Published:** 2025-10-05

**Authors:** Isaac Osei, Emmanuel Mendy, Kevin van Zandvoort, Olimatou Jobe, Golam Sarwar, Nuredin I. Mohammed, Jane Bruce, Ousman Barjo, Minteh Molfa, Rasheed Salaudeen, Brian Greenwood, Stefan Flasche, Grant A. Mackenzie

**Affiliations:** 1 Medical Research Council Unit, The Gambia at London School of Hygiene & Tropical Medicine, PO Box 273, Banjul, West Africa The Gambia; 2 Department of Disease Control, Faculty of Infectious and Tropical Diseases, London School of Hygiene & Tropical Medicine, London, UK; 3 Department of Infectious Disease Epidemiology, London School of Hygiene & Tropical Medicine, London, UK; 4 Centre for Mathematical Modelling of Infectious Diseases, London School of Hygiene & Tropical Medicine, London, UK; 5 Centre for Global Health, Charite– Universitätsmedizin, Berlin, Germany; 6 Murdoch Children’s Research Institute, Melbourne, Australia; 7 Department of Paediatrics, University of Melbourne, Melbourne, Australia

**Keywords:** *Streptococcus pneumoniae*, Vaccine-type, Infections, Contact, Pneumococcal conjugate vaccine

## Abstract

**Background:**

Although pneumococcal conjugate vaccines (PCVs) have been used widely in many low and middle-income countries, residual vaccine-type (VT) carriage persists in these settings. We examined the role of specific age groups in transmission and as reservoirs of VT pneumococcal infection in The Gambia.

**Methods:**

Between January and November 2022, we conducted a nested, population-based, cross-sectional social contact and pneumococcal carriage survey in the Central and Upper River Regions of The Gambia. Participants completed questionnaires on carriage risk factors and social contacts. Nasopharyngeal swabs were collected from selected household members across all age groups. *Streptococcus pneumoniae* was isolated and serotyped using standard methods. We analysed matched contact and pneumococcal carriage data and estimated the proportions of VT carriage attributable to contact with different age groups.

**Results:**

A total of 1638 participants were enrolled, of which 67% were children aged 0–14 years. Pneumococcal carriage prevalence was 59.6% (95% CI: 53.9 – 65.1%) in 0–4 year-olds and 36.1% (95% CI: 29.6 – 43.1%) in 5–14 year-olds. PCV13 VT carriage prevalence was not significantly different (10–13%) between these age groups. Among pneumococcal carriers, the proportion of VT carriage was significantly higher in 5- 14-year-olds [35.7% (95% CI: 25.9 – 46.9%)] compared to 0-4-year-olds [17.8% (95% CI: 13.9 – 22.6%, p-value < 0.001)]. The odds of VT carriage were 10% higher [AOR = 1.10, 95% CI: 1.01–1.20] for each additional physical contact with a child aged 10–14 years. We estimated that children aged 5–14 years contributed about 63% to the overall risk of exposure to VT pneumococci in the population.

**Conclusions:**

In rural Gambia, school-aged children, particularly those aged 5–9 years, are the main drivers of VT pneumococcal transmission. In the context of high coverage of routine PCV vaccination in infants, this suggests waning PCV protection by school entry. A booster dose for children at school entry may support better control of VT circulation in the population.

**Supplementary Information:**

The online version contains supplementary material available at 10.1186/s41479-025-00176-w.

## Introduction

In most low- and middle-income countries (LMICs), vaccine-type (VT) pneumococcal carriage persists despite prolonged use of pneumococcal conjugate vaccines (PCVs) at high coverage, in contrast to the situation observed in most high-income countries. For example, VT carriage prevalence among children aged < 5 years was substantially higher in The Gambia (17.5%) [1], Kenya (13.0%) [2], Mozambique (18.6%) [3], Malawi (18.7%) [4] and South Africa (15.6%) [5] as compared to the UK (0.7%) [6], Belgium (5.4%) [7], France (6.5%) [8] and the USA (4.8%) at similar post-vaccination time points [9]. The residual VT carriage in these countries may characterise a persistent reservoir population of VT pneumococci. This situation is a public health concern, as inadequate herd protection may result in a suboptimal reduction in disease burden.

It has been suggested that potential age-specific reservoirs within households and communities [10, 11], social contact patterns [12, 13], low vaccine coverage [14], and rapidly waning immunity [13] could each contribute to persistent VT carriage and transmission. There are limited data to support these assertions. Identifying potential transmission dynamics and sources of *Streptococcus pneumoniae* acquisition, including key drivers and reservoirs of infection, can help to disentangle the causes of persistent VT carriage in LMICs. While some studies have suggested that toddlers and infants are potential reservoirs of *S. pneumoniae* transmission within households [15, 16], other studies have indicated older children as the main reservoir in households and communities [11, 17–20], particularly in countries where high colonisation rates persist in older children.

There is evidence that close contact, especially physical contact, is implicated in the transmission of *S. pneumoniae* [19, 21, 22]. Of particular importance is the role that age groups and social contact patterns play in contributing to persistent VT carriage in high-transmission settings in the PCV vaccination era. We conducted a survey of social contacts in parallel with a population-based pneumococcal carriage survey in rural Gambia to examine the potential role of age groups and social contacts in the persistence of VT pneumococcal carriage.

## Methods

### Study setting and population

**Fig. 1:**
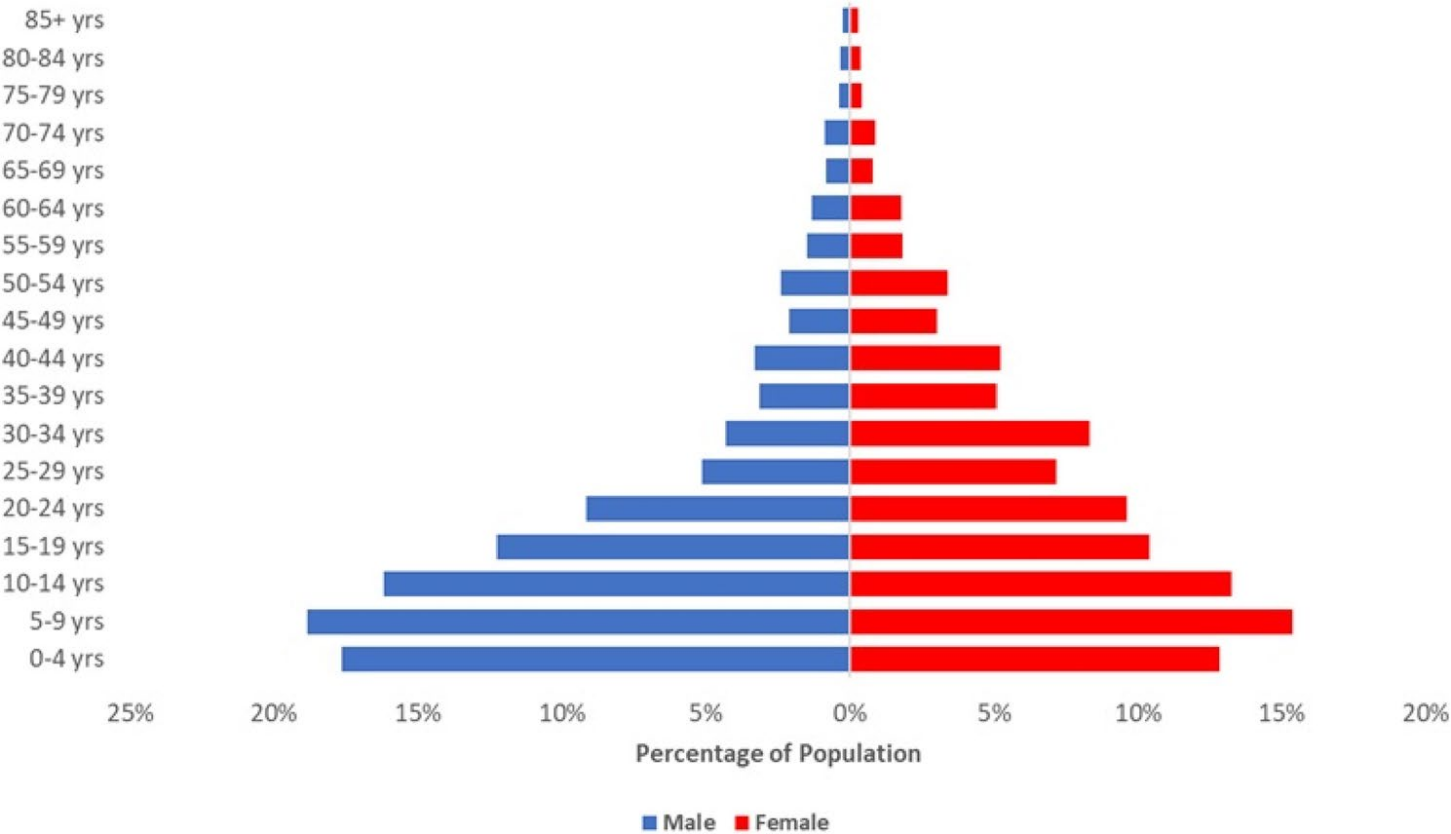
Age and sex distribution of the Basse Health and Demographic Surveillance System, December 2020.[27]

The social contact and carriage surveys were conducted in the Basse and Fuladu West Health and Demographic Surveillance Systems (BHDSS and FWHDSS) in Central River Region (CRR) and Upper River Region (URR), situated in the eastern part of The Gambia, respectively. In 2022, the population of the BHDSS was 206,429, distributed across 224 villages, and the FWHDSS population was 116,299, spread across 217 villages. In The Gambia, PCV is given as three primary doses scheduled at 2, 3, and 4 months of age (i.e. the standard ‘3p + 0’ schedule). Coverage rates for PCV are notably high, with 2018 estimates from the World Health Organization (WHO) and the United Nations Children’s Fund (UNICEF) indicating that 98% of infants received the first dose and 93% received the third dose [23, 24]. Similarly, in the study area, the coverage of three doses of PCV by 12 months of age was estimated to be 92% [25]. The BHDSS population is predominantly young, with approximately two-thirds of individuals below the age of 25 years and just 4% over 60 years. Children aged < 5 years constitute about 15% of the total population. The population has a higher number of females (54%) than males, with those aged 5–9 years being the most populous age group (Fig. 1). The FWHDSS population age structure is similar to the BHDSS’s. The study setting has been described previously [26–28].

### Study design

This study was nested within the Pneumococcal Vaccine Schedules (PVS) trial, which was conducted in the CRR and URR. PVS (trial number: ISRCTN15056916||http://www.isrctn.org/, registered on 15 November 2018) is a cluster-randomised trial that tested the non-inferiority of an alternative two-dose PCV schedule to the routine three-dose schedule. Between January and November 2022, we carried out a nested population-based, cross-sectional social contact survey within a larger pneumococcal carriage survey in the Central and Upper River Regions of The Gambia.

### Participant selection and sampling

**Fig. 2:**
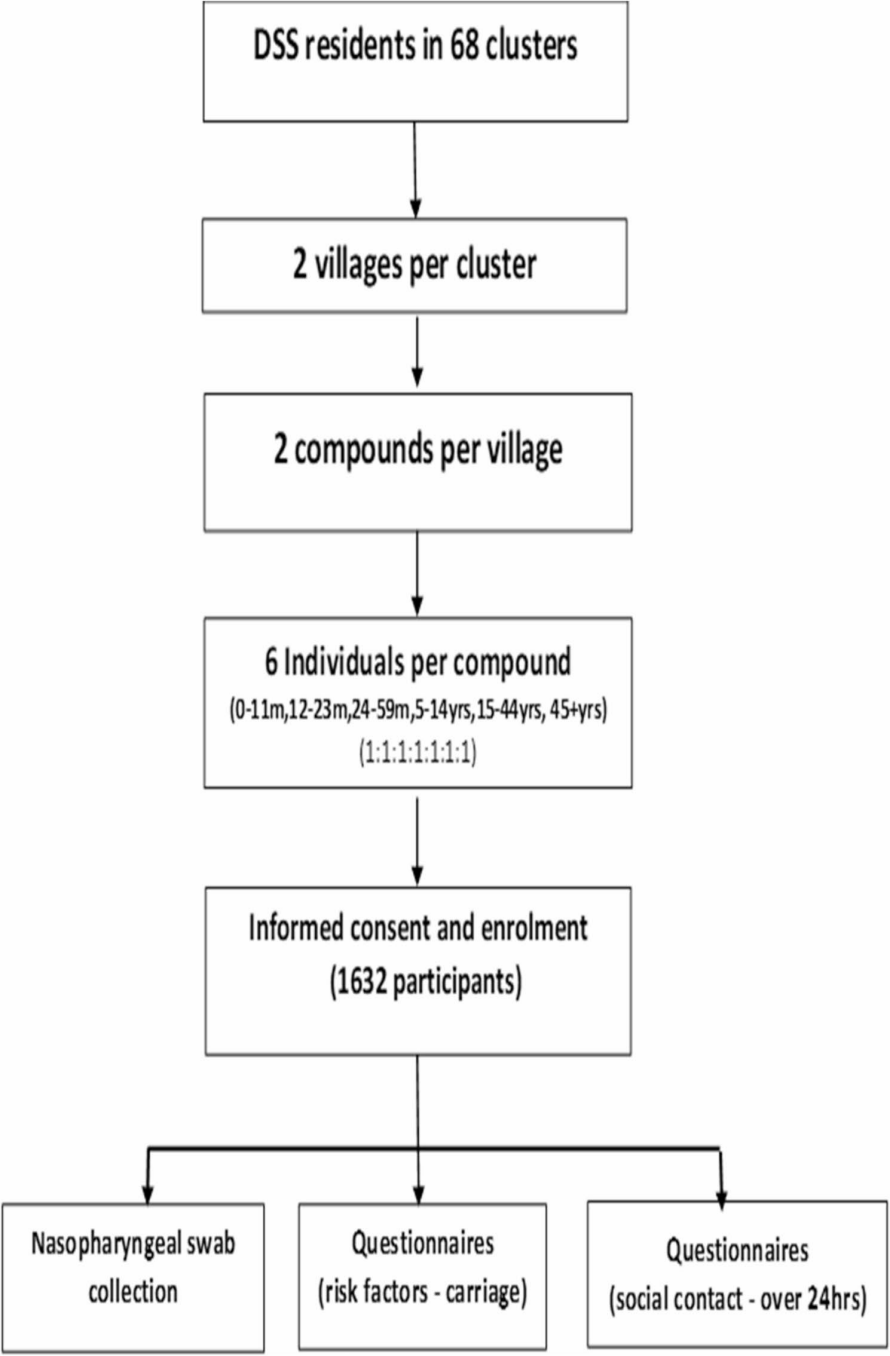
Flowchart of study design and sampling procedure

The sampling and participant selection processes for the social contact and carriage surveys have been described previously [29]. In summary, all residents from the 68 geographic clusters and 441 villages in the BHDSS and FWHDSS were eligible for enrolment. We probaly selected two villages from each cluster using probability proportional to size sampling. From each village, we randomly chose two compounds, and six individuals stratified by age group (0–11 months, 12–23 months, 24–59 months, 5–14 years, 15–44 years, and ≥ 45 years) were selected randomly from each compound. A minimum of 1632 participants were targeted for enrolment (Fig. 2).

### Data collection

The pneumococcal carriage survey used a standardised questionnaire [28], while the social contact questionnaire was adapted from an earlier contact survey [12, 29]. The procedure for collecting social contact and carriage data has been detailed elsewhere [28, 29]. In summary, at an initial visit, field workers informed potential participants about the study, obtained written informed consent from those willing to participate, explained the details of the social contact questionnaires, and primed them to make mental notes of their personal contacts the following day (24 hours). Parents or guardians gave consent for themselves and on behalf of child participants (under 18 years). Children aged 12 to 17 also provided assent. On the second visit (survey day), 48 hours after the first visit, the field worker took the participant through their activities in the previous 24 hours. The field worker asked for the initials and nicknames (if available) of the contacts, then went through all the listed contacts and selected participants to fill out a social contact questionnaire that detailed the number, intensity, location, regularity, duration, and age of interactions over the prior 24 hours. For children under 10 years old, a parent/guardian completed the social contact survey on their behalf, while parents assisted most older children in reporting their contacts. Participants provided estimated ages for non-household contacts. A contact (contactee) was defined as an individual whom the participant met in person during the 24 hours before waking up on the day of the survey with whom the participant had at least a short conversation and with whom they had either (i) “physical contact’’ (any skin-to-skin contact, e.g. a handshake, embracing, kissing, sleeping on the same bed, etc.), or (ii) “non-physical contact’’ (did not touch the person but exchanged at least a few words, face-to-face within two metres.”). Participants also provided estimates of the number of ‘casual contacts’ based on pre-determined age ranges (< 10, 10–19, 20–29, ≥ 30 years). We defined ‘casual contacts’ as brief interactions lasting under five minutes. We collected information on participants’ demographics, vaccination history, and risk factors for carriage during the second visit. Data were collected electronically using a customised application. The detailed findings from the social contact study [29] and the larger pneumococcal carriage survey [28] have been previously reported.

### Laboratory methods

The laboratory procedures for culturing and identifying *S. pneumoniae* have been documented previously [28]. In summary, a single nasopharyngeal specimen per participant was collected using nylon-tipped, flexible, nasopharyngeal flocked swabs following WHO guidelines [30]. The swabs were immediately inserted into a vial of skim-milk tryptone glucose glycerol (STGG) transport medium. The packaged sample was placed between two inner layers of foam in a specimen cold box, transported within 8 h of collection, and stored at −80 °C at the Basse Medical Research Council laboratory. *S. pneumoniae* was identified by alpha haemolytic colony morphology on blood agar, susceptibility to optochin and bile solubility. A single colony, or any colonies that exhibited different morphological characteristics, were selected from each plate for sub-culturing and serotyping. We employed a standardized latex agglutination method to serotype each isolate and used the Quellung reaction to confirm serotyping when necessary.

### Statistical power and sample size

A minimum of 1632 participants were selected to provide 90% power to detect an absolute difference of at least one mean contact per day between age groups. This sample size calculation was based on the findings of a social contact survey conducted in rural Uganda [29, 31].

### Statistical considerations

A descriptive analysis of background characteristics and risk factors for carriage was conducted. Age-specific probability weights were applied to account for oversampling in the younger age group at the design stage, enabling the calculation of population-level estimates. We employed design weights to account for clustering. Age-stratified contact matrices, which examined physical and non-physical contact patterns, were computed using the socialmixr [32] package in R. These matrices incorporated post-stratification weights based on age, gender, and weekday and were adjusted for the reciprocity of contacts. Each matrix element (*mij*) represents the estimated mean number of contacts between individuals in age group *i* and those in age group *j*, while *wi* indicates the population size in age group *i*. During the initial analysis, we examined the associations between contact frequency, contact type (physical or non-physical), location, duration, and regularity with pneumococcal nasopharyngeal and PCV13 VT carriage. This analysis was performed using univariable Generalized Estimating Equations (GEEs) with a log link, binomial family, and exchangeable correlation structure, adjusted for clustering at the cluster level. The outcome variables, namely pneumococcal and VT carriage, were considered binary. Point estimates were presented for both unadjusted and age and gender adjusted odds ratios along with a 95% confidence interval (95% CI). In the second stage of analysis, we modelled the association between VT carriage and the number of contacts per 24 h by contact type (physical or non-physical) made with specific age-stratified groups using a logistic GEE model adjusted for age and gender and accounting for clustering. We performed logistic regression analysis to examine risk factors associated with overall pneumococcal and VT carriage.

#### Determining specific age-group contribution to VT pneumococcal exposure

To investigate the potential contribution of various age groups to VT pneumococcal exposure, we combined contact patterns with VT carriage prevalence data to estimate exposure across age groups using a similar method to that employed by Qian et al. [20]. This approach calculates the risk for at least one exposure on a given day as the risk for at least one contact with an individual who is currently colonized with pneumococci. The age-specific VT prevalence within a particular age group acts as a proxy for the probability that contact with an individual in that group leads to pneumococcal exposure. This approach assumes that such exposure relies solely on the age group involved. Utilizing the linked dataset, we determined the percentage of VT carriage exposure in an age group attributable to contact with either the same or another specific group. An age group that contributed above the average to overall VT pneumococcal exposure, in comparison to others, was classified as a reservoir for VT pneumococcal carriage exposure for that group. We calculated 95% CIs around the exposure estimates by bootstrapping the mean contact rates between age groups and the prevalence estimates within each age group. This analysis was conducted in R version 4.2.2 [33].

### Ethical approval

The study was approved by the Gambia Government/MRC Joint Ethics Committee (ref: 28705) and the LSHTM Ethics Committee (ref: 28705).

## Results

### Characteristics of the study participants

A total of 1,638 participants from 611 households living in 158 compounds across 441 villages participated in the pneumococcal carriage and contact survey. One individual declined consent, and there were two missing nasopharyngeal swab results. Approximately 50% of the participants were under 5 years of age. More females (54%) were enrolled compared to males, with 54% of the data collected during the dry season, and 88% of those under 10 years had received at least two doses of PCV13 (vaccinated). About half of the participants (49%) lived in compounds comprising 20 to 49 members, and 48% of participants shared a bed with a child. More than half (66%) had experienced upper respiratory tract infection symptoms in the previous two weeks, and a quarter of the participants had taken antibiotics within a fortnight prior to the survey day (Table 1). The age and sex distribution of enrolled participants are shown in Supplementary Figure 1.

**Table 1:** Socio-demographic characteristics of participants in a cross-sectional carriage and contact survey in the gambia, 2022 (*N* = 1,638)

Characteristics		*n* (%)
Age in months (m) or years (yrs)		
	0–11 m	277 (16.9)
	12–23 m	271 (16.5)
	24–59 m	272 (16.6)
	5–9 yrs	149 (9.1)
	10–14 yrs	130 (7.9)
	15–44 yrs	269 (16.4)
	≥ 45 yrs	270 (16.5)
Sex		
	Male	747 (45.6)
Ethnicity		
	Fula	978 (59.8)
	Serahule	226 (13.8)
	Mandinka	312 (19.1)
	Wolof	110 (6.7)
	Others	12 (0.7)
Season		
	Dry	881 (53.8)
^a,b^PCV13 vaccinated		
	Yes	803 (88.6)
^c^Compound size		
	1–19	507 (30.9)
	20–49	795 (48.6)
	≥ 50	336 (20.5)
Number of enrolled participants per household		
	1–3	403 (66.0)
	4–5	157 (25.7)
	6–7	51 (8.3)
Bed-sharing with a child aged < 10 yrs		
	Yes	791 (48.3)
^*^Cooking method		
	Firewood	1,618 (98.8)
	Charcoal/Other	11 (0.7)
^*^Cooking location		
	Inside	1,006 (61.4)
	Outside	51 (3.1)
	Inside & outside	574 (35.0)
^*^Smoker in the household		
	Yes	607 (37.0)
^*^Has had a runny nose within the past two weeks		
	Yes	1,086 (66.3)
Took antibiotics in the last two weeks		
	Yes	394 (24.0)

*Missing values; cooking location = 7, cooking method = 9, Has had a runny nose within the past two weeks = 7

^a^Those who received at least two doses of PCV - calculated in children < 10 years

^b^We could not validate the PCV status of 6.4% (62/968) of children < 10 years

^c^A compound consists of groups of families typically residing together in a living arrangement

#### Age-specific contact patterns

**Fig. 3:**
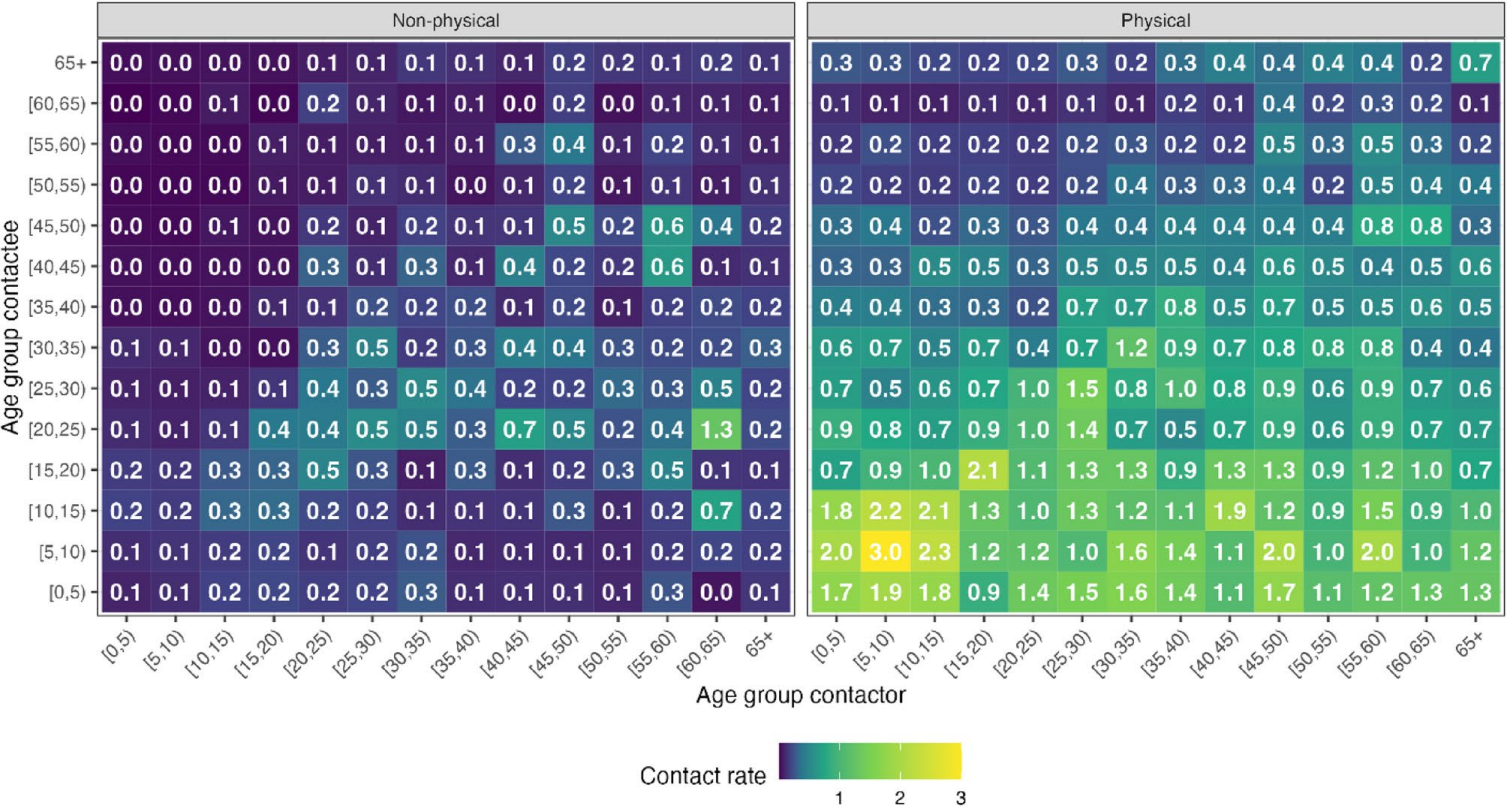
Contact matrices. The weighted mean number of daily non-physical and physical contacts made by respondents with contacts of different age groups. Parents and caregivers acted as proxies to estimate contacts of young children < 10 years. Both matrices are adjusted for the reciprocity of contacts

The majority of the daily average number of contacts per person was physical. Most physical contacts made by participants of any age occurred with children, highlighting the youthful age structure of the population in this setting [27]. Physical contact patterns were predominantly age assortative, as evidenced by the high contact rates along the diagonal of the matrix up to the age group of 25–30 years, suggesting that individuals in the population were more likely to engage with others of a similar age group (fig 3). The highest daily mean physical contact rates were observed among children aged 5-10 years, while the lowest rates within age group contacts were noted among those aged 50–55 years and 60-65 years. Older children reported high rates of physical contact with infants and toddlers under 5 years old and fewer physical contacts with adults over 25 years old. The highest daily mean non-physical contact rates were recorded among those aged 60-65 and those aged 20-25 years (fig 3).

#### Pneumococcal carriage

The age-adjusted overall estimated prevalence of pneumococcal carriage was 27.8% (95% CI: 23.8% – 32.1%). The age-adjusted overall prevalence of VT carriage was 6.7% (95% CI: 4.9%−8.9%). Approximately 2.2% of all pneumococci were non-typeable isolates, while the prevalence of non-PCV13 serotypes was 18.7% (95% CI: 16.2% - 21.5%, Table 2). The prevalence of VT carriage in the 0-4 years age group was 10.6% (95% CI: 8.2% - 13.7%) with similar prevalence in the age-groups 0-11 months, 12-23 months, and 2-4 years. Vaccine-type carriage prevalence was 13.0% (95% CI: 9.2% - 18.0%) in the 5-14 years age group, 14.9% (95% CI: 9.6% - 22.5%) among 5-9 year-olds and 10.8% (95% CI: 6.1% – 18.3%) in 10-14 year olds. Although the 13.0% prevalence of VT carriage among those aged 5-14 years was higher than the 10.6% among 0-4-year-olds, the difference was not statistically significant (p-value = 0.27, Table 2). Similarly, although the 14.9% prevalence of VT carriage among 5-9-year-olds was higher than the 10.6% in those aged 0-4 years, this difference was not statistically significant (p-value = 0.13). Among pneumococcal carriers, the proportion of VT carriage was significantly higher in 5-14-year-olds [35.7% (95% CI: 25.9%– 46.9%)] compared to 0-4-year-olds [17.8% (95% CI: 13.9%– 22.6%, p-value < 0.001)]. An increase in age and the use of antibiotics within two weeks prior to the survey were associated with reduced odds of both overall pneumococcal and VT carriage. In contrast, males and the dry season were linked to a higher likelihood of pneumococcal and VT carriage (Tables 5 & 7 in the Supplementary Material).

**Table 2:** Pneumococcal carriage prevalence by specific age groups and proportions of different Pneumococcal vaccine types among carriers

Overall		*N* = 1636		^a^Population-level Prevalence (%) (95% CI)	^a^Proportion among carriers (%) (95% CI)
		VT (*n* = 135)		6.7 (4.97–8.89)	24.0 (18.85–30.07)
		NVT (536)		18.8 (15.98–22.12)	67.9 (61.62–73.63)
		NT (33)		2.2 (1.25–3.97)	8.1 (4.62–13.71)
		Any Spn (704)		27.8 (23.80–32.14)	
Age in months (m) or years (yrs)					
	0–11 m	*N* = 277			
		VT (*n* = 31)		11.2 (8.19–15.11)	15.4 (11.31–20.68)
		NVT (163)		58.8 (52.37–65.03)	81.1 (74.97–86.00)
		NT (7)		2.53 (1.10–2.97)	3.5 (1.52–7.76)
		Any Spn (201)		72.6 (66.32–78.03)	
	12–23 m	*N* = 271			
		VT (27)		9.9 (6.95–14.09)	14.4 (10.37–19.74)
		NVT (156)		57.6 (51.65–63.27)	83.4 (77.97–87.73)
		NT (4)		1.5 (0.55–3.88)	2.1 (0.80–5.56)
		Any Spn (187)		69.0 (62.07–75.17)	
	24–59 m	*N* = 272			
		VT (29)		10.7 (7.31–15.30)	20.0 (13.80–28.07)
		NVT (109)		40.1 (33.04–47.54)	75.2 (65.93–82.57)
		NT (7)		2.6 (1.01–6.37)	4.8 (1.92–11.60)
		Any Spn (145)		53.3 (45.91–60.57)	
	^#^0–4 yrs	*N* = 820			
		VT (87)		10.6 (8.18–13.71)	17.8 (13.88–22.62)
		NVT (428)		46.6 (41.38–51.89)	78.2 (72.79–82.77)
		NT (18)		2.4 (1.19–4.67)	4.0 (2.01–7.69)
		Any Spn (533)		59.6 (53.87–65.08)	
	5–9 yrs	*N* = 147			
		VT (22)		14.9 (9.66–22.46)	40.7 (27.72–55.20)
		NVT (29)		19.7 (13.49–27.92)	53.7 (38.99–67.79)
		NT (3)		2.0 (0.66–6.17)	5.6 (1.75–16.28)
		Any Spn (54)		36.7 (28.61–45.69)	
	10–14 yrs	*N* = 130			
		VT (14)		10.8 (6.10–18.30)	30.4 (17.26–47.85)
		NVT (27)		20.8 (13.72–30.18)	58.7 (41.50–73.99)
		NT (5)		3.8 (1.61–8.89)	10.9 (4.63–23.43)
			Any Spn (46)	35.4 (27.10–44.65)	
	^#^5–14 yrs	*N* = 277			
		VT (36)		13.0 (9.25–18.01)	35.7 (25.92–46.87)
		NVT (56)		20.2 (15.14–26.45)	56.1 (45.01–66.68)
		NT (8)		2.9 (1.50–5.65)	8.1 (4.31–14.87)
		Any Spn (100)		36.1 (29.58–43.09)	
	15–44 yrs	*N* = 269			
		VT (6)		2.2 (0.88–5.49)	13.9 (5.69–30.33)
		NVT (31)		11.5 (7.89–16.53)	72.1 (56.11–83.92)
		NT (6)		2.2 (0.89–5.49)	13.9 (5.81–29.87)
		Any Spn (43)		15.9 (11.39–21.96)	
	≥ 45 yrs	*N* = 270			
		VT (6)		2.2 (1.01–4.82)	21.4 (10.28–39.36)
		NVT (21)		7.8 (5.24–11.39)	75.0 (56.83–87.24)
		NT (1)		0.4 (0.05–2.68)	3.6 (0.47–22.44)
		Any Spn (28)		10.4 (7.34–14.46)	

*NVT* Non-PCV13 serotypes

*NT* Non-typeable pneumococci

*Any Spn* Any pneumococcal serotype, including non-typeable

^a^Estimates are the weighted proportions and prevalence with 95% CIs adjusted for clustering at the cluster level

^#^Aggregated estimates in those aged < 5 years and those aged 5–14 years

#### Association between contact parameters and overall pneumococcal and VT carriage

The number of household contacts showed a significant association with the risk of pneumococcal carriage [Adjusted Odds Ratio (AOR), 1.11 (95% CI: 1.03–1.19, *p* < 0.01), Table 4 in the Supplementary Material]. The frequency of contact, contacts with familiar individuals, and contacts lasting longer than one hour were associated with an increased odds of pneumococcal carriage; however, these associations were not statistically significant. In comparison to non-physical contact, there was some evidence of an association between physical contact and VT carriage [AOR, 1.22 (95% CI: 1.00–1.48, *p* = 0.05)]. The odds of VT carriage were 10% higher [AOR, 1.10 (95% CI: (1.01–1.20)] for each additional physical contact with a child aged 10 - 14 years (Table 3).

**Table 3:** Association of contact parameters with PCV13 vaccine type (VT) nasopharyngeal carriage

Contact characteristics	Number of contacts *n* (%)	Crude Odds ratio (95% C1)		*p*-value	Adjusted Odds ratio^a^(95% C1)	*p*-value
*Frequency of contacts*						
Total number of contacts per 24 h	19,811 (100)	0.99 (0.95–1.03)		0.65	1.01 (0.97–1.05)	0.78
*Contact type*						
Non-physical	2,904 (14.6)	1 (ref)			1 (ref)	
Physical contact	16,908 (85.4)	1.24 (1.02–1.50)		0.03	1.22 (1.00–1.48)	0.05
*Location*						
Non-household	3,686 (18.6)	1 (ref)				
Household	16,125 (81.4)	0.96 (0.76–1.19)		0.69	0.91 (0.73–1.14)	0.43
*General Regularity*						
Infrequent	2,823 (14.3)	1 (ref)			1 (ref)	
Daily/almost daily	16,922 (85.7)	1.31 (0.98–1.75)		0.07	1.26 (0.95–1.69)	0.11
*Duration*						
Contact < 1 h	2,968 (15.0)	1 (ref)			1 (ref)	
Contact ≥ 1 h	16,763 (85.0)	1.51 (0.98–2.32)		0.06	1.45 (0.94–2.26)	0.09
*Number of physical contacts per 24 h made with those*:						
< 2 years	1,052 (6.2)	0.90 (0.65–1.25)		0.55	0.93 (0.68–1.27)	0.64
2–4 years	1,490 (8.8)	1.18 (1.00–1.39)		0.04	1.12 (0.95–1.32)	0.17
5–9 years	3,155 (18.7)	1.05 (0.93–1.17)		0.45	1.01 (0.88–1.12)	0.87
10–14 years	2,564 (15.2)	1.11 (1.01–1.23)		0.03	1.10 (1.00–1.21)	0.05
15–44 years	6,675 (39.5)	0.92 (0.85–1.00)		0.05	0.95 (0.87–1.03)	0.23
45 + years	1,951 (11.6)	0.91 (0.73–1.14)		0.41	0.99 (0.76–1.30)	0.96
^ #^0–4 years	2,542 (15.0)	1.06 (0.93–1.19)		0.34	1.04 (0.92–1.17)	0.56
^ #^5–14 years	5,719 (33.9)	1.06 (0.99–1.12)		0.07	1.03 (0.97–1.10)	0.38

^a^Adjusted for age, gender, season, and antibiotic use

^#^Age-group of interest

#### Specific age-group contribution to VT pneumococcal exposure

**Fig. 4:**
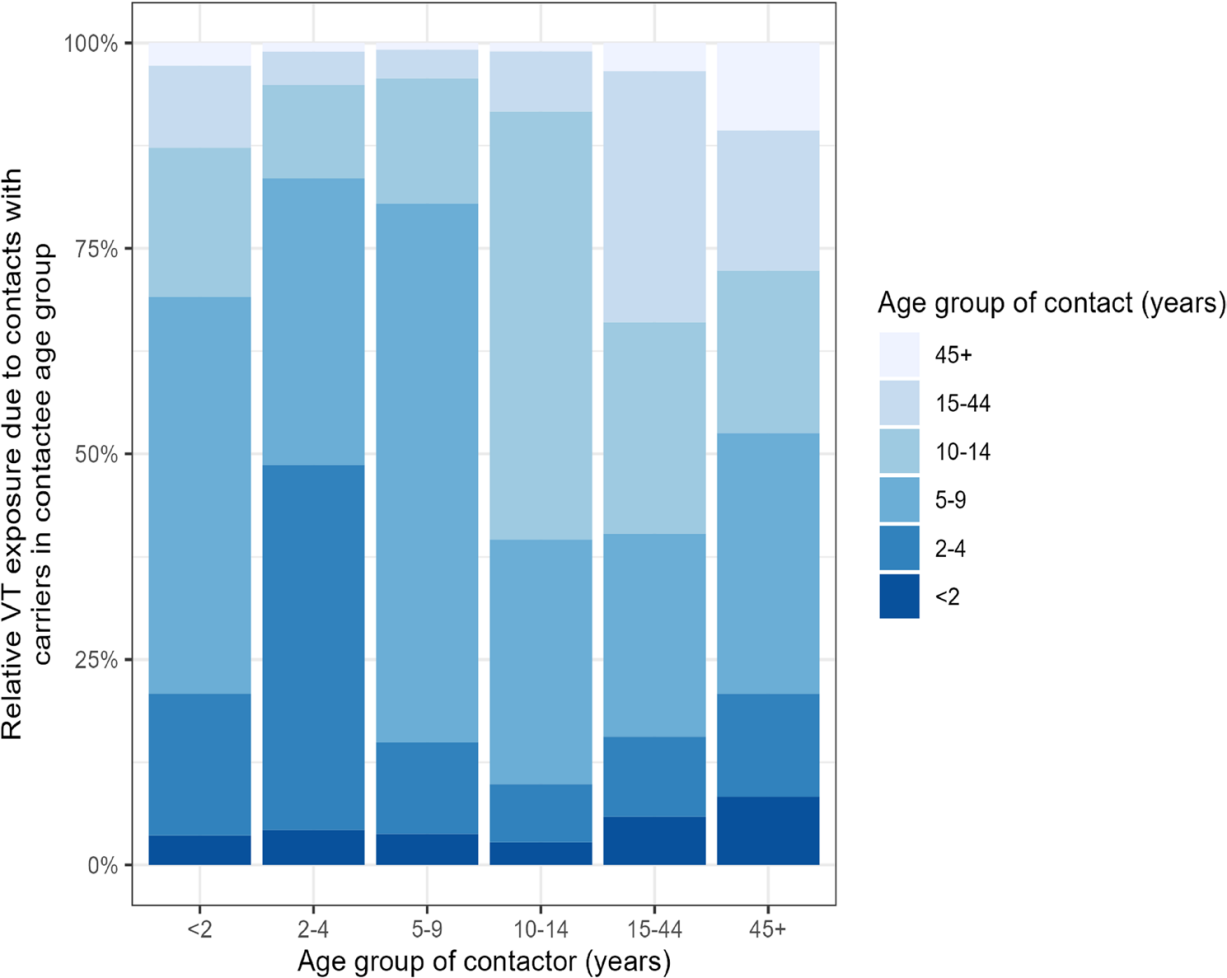
The contribution of different age groups towards the total exposure of VT pneumococci of other age groups. The bars show the relative proportions of VT exposure due to contacts with carriers in the contactee age group

Due to a limited number of contacts, the age groups 0-11 months and 12-23 months were combined into a single category. A significant proportion (49% (95% CI: 35% - 60%)) of VT exposure to children under 2 years old may be linked to contacts with children aged 5-9 years, followed by equal contributions from children aged 2-4 years [17% (95% CI: 11% - 25%)] and those aged 10-14 years [17% (95% CI: 9% - 29%)]; see Fig. 4. Children aged 2-4 years [44% (95% CI: 29% - 57%)], 5-9 years [65% (95% CI: 52% - 76%)], and 10-14 years [52% (95% CI: 33% - 68%)] represented the primary sources of VT pneumococcal exposures within their respective age groups. Children aged 5-9 years [25% (95% CI: 15% - 38%)], 10-14 years [26% (95% CI: 14% - 40%)], and those aged 15-44 years [29% (95% CI: 11% - 47%)] contributed similarly to the overall VT pneumococcal exposure in the 15-44 years age group. Those aged 5-9 years were the primary source of VT pneumococcal exposure for individuals aged ≥ 45 years, contributing the most [32% (95% CI: 21% - 43%)] to VT pneumococcal exposure compared to other age groups. Although VT carriage prevalence was relatively high among children under 2 years old, their contribution to VT pneumococcal exposure in the population was < 5%. Similarly, those aged 45 years or older contributed < 5% to the overall VT pneumococcal carriage exposure within their group and to other age groups as well. Those aged 5-9 years were the key drivers of VT pneumococcal transmission in three of the six age groups in this study; that is, within their age group [65% (95% CI: 52% - 76%)], as well as among the < 2-year-olds [49% (95% CI: 35% - 60%)] and the older age group, aged ≥45 [32% (95% CI: 21% - 43%)]. (Supplementary Table 6).

## Discussion

Using matched social contact and age-specific pneumococcal carriage prevalence data, we examined the potential role of age groups and social contacts in persistent VT pneumococcal carriage. We found evidence that, compared to non-physical contact, physical contact was associated with higher odds of VT pneumococcal carriage. This finding supports the existing evidence that physical contact is relevant to pneumococcal transmission [19–22]. We found that children aged 5 to 14 years contributed approximately 63% to the overall risk of exposure to VT pneumococci in the population. In particular, those aged 5-9 years were identified as key drivers of VT pneumococcal transmission in three of the six age groups in this study. The high prevalence of VT carriage and the elevated rates of physical contact observed among school-aged children may account for this finding.

Research on nested social contacts and pneumococcal carriage is limited. In contrast to our findings, a similar survey conducted in Nha Trang, Vietnam, aimed at investigating pneumococcal transmission routes to infants, identified 1-4-year-olds as the primary transmitters of pneumococcal infection to infants, although 5-9-year-olds also contributed modestly [20]. In line with the Vietnamese study, we found robust evidence that the odds of pneumococcal carriage increased by 1.11 (95% CI: 1.03–1.19) for each additional household contact. In their study in Southwest Uganda, Le Polain de Waroux et al. also found evidence that the number of household contacts increased the risk of pneumococcal carriage [22]. However, a similar infant contact mixing study in the UK found the majority of contacts with infants to be non-household [34]. This highlights the important role of the local context in the transmission routes of various infections that spread through close contact. We did not find a significant association between VT carriage and household contact. We observed a high rate of physical contact between older children aged 10 - 14 years and infants. This is in contrast to a study in Fiji, which found a low rate of contact between older children and infants [21]. Many LMICs have a young population age distribution similar to The Gambia's [35], and when considered at the community level, the age group reservoir of pneumococci and the likely transmission pattern could be markedly influenced by the population's age distribution [36]. In our setting, older siblings of infants, including those who are school-aged, typically share childcare responsibilities [37]. These unique local contextual factors may explain our finding that 5- 14-year-olds are the primary transmission group for infant pneumococcal infection.

We found that the odds of VT carriage were 10% higher for each additional physical contact with children aged 10 - 14 years. This aligns with our findings in a related study, which indicated that despite the high coverage of PCV vaccination in this setting, among PCV-vaccinated children under 10, the odds of PCV13 VT carriage in 5–9-year-olds were 1.60 times higher than in infants [38]. It also supports the observation that those aged 10 - 14 years contributed modestly to the VT pneumococcal exposure to other age groups. Our results are consistent with a longitudinal cohort study involving the Navajo Nation and White Mountain Apache tribes, where a probability transmission model suggested that contact with older children was linked with pneumococcal transmission and acquisition in younger children [11]. This evidence further suggests that the protection offered by PCV has likely waned in this age group, reinforcing the consideration for catch-up vaccinations for older children.

As noted, despite the relatively high prevalence of VT carriage in infants and toddlers, their contribution to VT pneumococcal exposure in other age groups was minimal. As previously reported, children < 2 years old had few social contacts [29]. Their low contact rate with individuals from different age groups may explain their limited contribution to VT pneumococcal exposure in other age groups. Our finding is consistent with a cross-sectional study on social contact and pneumococcal carriage among internally displaced people in Somaliland. This study showed that although pneumococcal carriage prevalence was relatively high in children < 2 years, they contributed little to the onward transmission to other age groups due to their limited social contacts [19].

Although children aged 5-9 years represent 15% of the population, the difference in VT carriage rates between this age group (VT carriage prevalence of 15%) and those over 15 years, who account for approximately 55% of the population (VT carriage prevalence of 2.2%), is notably more pronounced. Furthermore, the proportion of VT carriage among pneumococcal carriers within the 5-9 year old group (41%) is significantly higher than in any other age group, demonstrating robust circulation of VT carriage within this demographic. Identifying school-aged children, particularly those aged 5-9 years, as the key drivers of VT pneumococcal transmission in rural Gambia may have implications for PCV vaccination strategies. The high prevalence of VT carriage observed among school-aged children, despite the approximately 90% PCV vaccination coverage in children under 10 years in this study, suggests that the protection provided by PCV has likely waned in this age group. This finding highlights the importance of considering catch-up vaccinations for school-aged children and advocates for the possible addition of booster doses to PCV schedules.

Our study has several strengths. First, our sample size is substantial and includes individuals of all age groups. Similar studies conducted in other settings had either a smaller sample size [12, 19] or did not include specific age groups [21]. Second, data on contacts were collected on the same day that nasopharyngeal samples were collected, which enabled a plausible linkage of an individual's contact behaviour to the risk of pneumococcal carriage acquisition. Third, our data collection spanned the two main seasons in The Gambia, enabling seasonal variation to be measured. Lastly, we were able to serotype a high percentage of isolated pneumococcal samples (~2% were non-typeable).

Notwithstanding, there are some limitations to our study. The contact survey is prone to recall bias as participants may not be able to recall all their contacts for the past 24 hours. This may result in misclassification of reported contact. To mitigate a potential bias in the data collected on contacts, we conducted two visits within 72 hours; during the first visit, we collected household demographic information and encouraged participants to take mental notes of their contacts. The second visit focused on collecting the recalled contact information. Studies have shown that the accuracy of reporting increases when individuals are prompted in advance to note their interactions [39]. Furthermore, employing sensitive techniques like microarray and polymerase chain reaction (PCR) would have enhanced the detection of *S. pneumoniae* carriage in comparison to traditional culture methods. Adoption of a longitudinal household study would have enabled direct observation of transmission within and between age groups. Longitudinal household studies to detect the direction of pneumococcal transmission events require intensive, frequent sampling of all household members, with the origin of some acquisition events being unobserved. The role of contacts outside the household may be challenging to measure in such studies. While our study was not designed to establish causality, nested cross-sectional contact and pneumococcal carriage surveys in Vietnam, Fiji, Somaliland, and Uganda demonstrated that the combination of social contact and age-dependent infection probabilities offers a simple and suitable alternative method of identifying pneumococcal transmission dynamics [20, 22]. Although the assumptions regarding the consistency of behaviour between when social contact data is collected and when the infection outcome is observed can be challenging to validate, studies have shown that an individual social contact pattern is unlikely to be influenced by their pneumococcal carriage status, as the majority of carriage remains asymptomatic. The correlation between contact behaviour and pneumococcal carriage status observed in this study and previous studies suggests that contact patterns recorded on a specific day are a reasonable reflection of an individual's overall contact behaviour, more so around the time of pneumococcal acquisition [20–22, 40, 41].

## Conclusion

We found evidence that physical contact significantly contributes to the transmission of VT *S. pneumoniae*. We observed that in rural Gambia, with sustained high coverage of PCV for many years, children aged 5-14 years, particularly those aged 5-9 years, are the main reservoir for continued VT pneumococcal exposure to the younger age group and older age groups. Despite high PCV vaccination rates, the high VT carriage in school-aged children suggests that the protection from PCV has at least partly waned in this age group. Our results provide insights to inform alternative dosing strategies and vaccination schedules for PCVs, especially the inclusion of a school entry booster dose in high-transmission settings.

## Supplementary Information

Supplementary Material 1.

## Data Availability

Contact data are available on Zenodo via 10.5281/zenodo.13101862, and the questionnaires are available at 10.5281/zenodo.14064156.

## Abbreviations

BHDSS: Basse Health and Demographic Surveillance System
FWHDSS: Fuladu West Health and Demographic Surveillance Systems
DSS: Demographic Surveillance System
PVS: Pneumococcal Vaccine Schedules trial
PCV: Pneumococcal Conjugate Vaccine
NPS: Nasopharyngeal Swab
VT: Vaccine-type Serotype
LMICs: Low and Middle Income Countries
WHO: World Health Organisation
UNICEF: United Nations International Children's Fund
