# Supplementary material for: Identification of age-group reservoirs for persistent vaccine-type pneumococcal carriage in rural Gambia
Source: Pneumonia (Nathan). 2025 Oct 5;17:22. doi: 10.1186/s41479-025-00176-w (PMC12497339; doi:10.1186/s41479-025-00176-w)
Supplement: Supplementary file 1 — Supplementary Material 1. [file 41479_2025_176_MOESM1_ESM.docx]

Supplemental Material for

**Identification of specific age-group reservoirs for persistent vaccine-type pneumococcal carriage in rural Gambia**

Isaac Osei^*^, Emmanuel Mendy, Kevin van Zandvoort, Olimatou Jobe, Golam Sarwar, Nuredin I Mohammed, Jane Bruce, Ousman Barjo, Minteh Molfa, Rasheed Salaudeen, Brian Greenwood, Stefan Flasche, Grant A Mackenzie

* Corresponding author: Isaac Osei, [Isaac.Osei@lshtm.ac.uk](mailto:Isaac.Osei@lshtm.ac.uk)

**Table of Contents**

[SF D1: Age and sex distribution of participants enrolled in a cross-sectional social contact and neumococcal carriage surveys conducted in 2022 (N = 1,638) 3](#_Toc200321093)

[SF D2: Distribution of runny noses and antibiotic use among age groups participating in a cross-sectional survey of social contacts and pneumococcal carriage conducted in 2022 (N = 1,638) 4](#_Toc200321094)

[ST D1: Association of age groups of participants and contactees with contact duration 5](#_Toc200321095)

[ST D2: Association of age groups of participants and contactees with contact location 6](#_Toc200321096)

[ST D3: Association of age groups of participants and contactees with the general regularity of contact 7](#_Toc200321097)

[ST D4: Association of contact parameters with overall pneumococcal nasopharyngeal carriage 8](#_Toc200321098)

[ST D5: Unadjusted and adjusted odds ratios showing the association of risk factors with any pneumococci nasopharyngeal carriage in a cross-sectional carriage and contact survey conducted in 2022 (n = 1636) 9](#_Toc200321099)

[ST D6: The contribution of different age groups toward age-specific exposure to vaccine-type pneumococcus 11](#_Toc200321100)

[ST D7: Unadjusted and adjusted odds ratios showing the association of risk factors with PCV13 vaccine-type pneumococcal nasopharyngeal carriage in a cross-sectional carriage and contact survey conducted in 2022 (n = 1636) 12](#_Toc200321101)

**SF = Supplementary Figure**

**ST = Supplementary Table**

# **SF D1: Age and sex distribution of participants enrolled in a cross-sectional social contact and neumococcal carriage surveys conducted in 2022 (N = 1,638)**


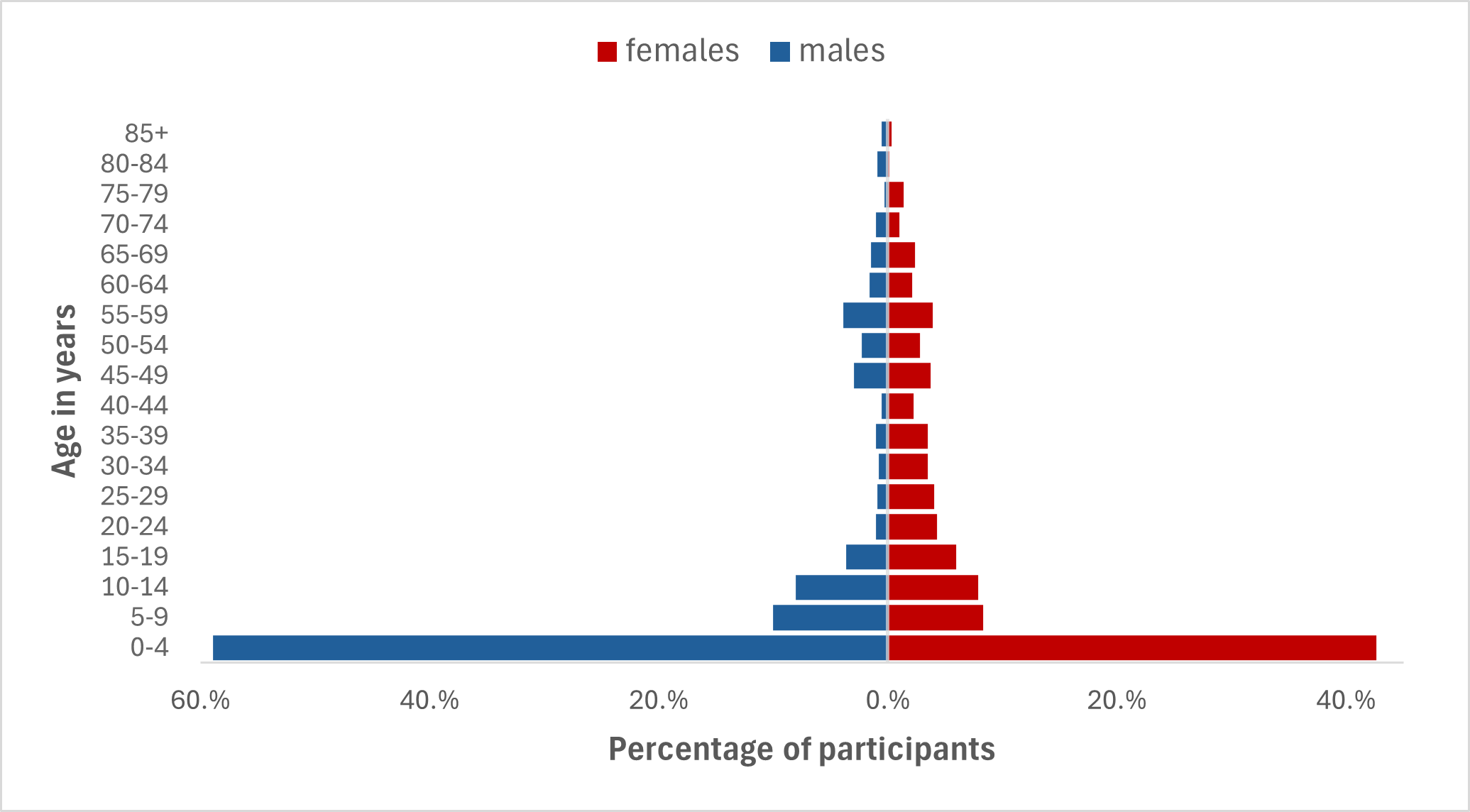


# **SF D2: Distribution of runny noses and antibiotic use among age groups participating in a cross-sectional survey of social contacts and pneumococcal carriage conducted in 2022 (N = 1,638)**


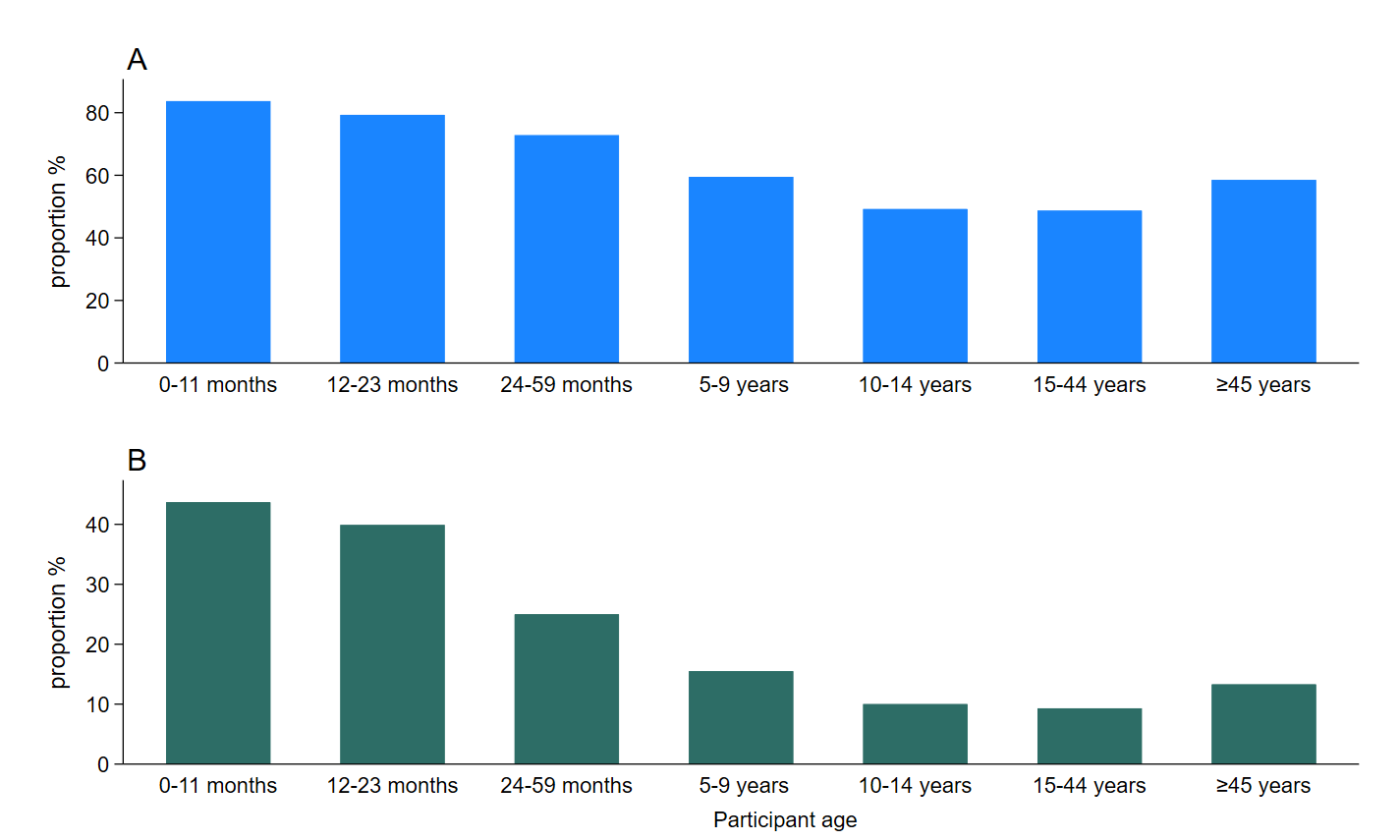


**(A)** The proportion of participants who reported experiencing a runny nose in the two weeks preceding the survey, categorised by age group. **(B)** The proportion of participants who reported having used antibiotics in the two weeks preceding the survey, categorised by age group.

# **ST D1: Association of age groups of participants and contactees with contact duration**

| Age Category | Number of contacts lasting ≥ 1 hour  n (%) | Adjusted Odds ratio^a^  (95% C1) | p-value |
| --- | --- | --- | --- |
| *Participant age group in months (m) or years (yrs)* |  |  |  |
| 0-11 m | 2,641 (86.7) | 1 (ref) |  |
| 12-23 m | 2,658 (88.2) | 1.14 (0.88 – 1.48) | 0.31 |
| 24-59 m | 2,769 (87.5) | 1.07 (0.85 – 1.34) | 0.58 |
| 5-9 yrs | 1,598 (84.9) | 0.85 (0.63 – 1.16) | 0.31 |
| 10-14 yrs | 1,490 (88.4) | 1.16 (0.83 – 1.62) | 0.39 |
| 15-44 yrs | 2,820 (82.3) | 0.69 (0.52 – 0.90) | <0.01 |
| ≥ 45 yrs | 2,787 (79.3) | 0.58 (0.45 – 0.73) | <0.001 |
| Age group of contacts in months (m) or years (yrs) |  |  |  |
| 0-11 m | 349 (97.8) | 1 (ref) |  |
| 12-23 m | 743 (94.9) | 0.53 (0.21 – 1.38) | 0.19 |
| 24-59 m | 1,441 (91.4) | 0.35 (0.16 – 0.79) | 0.01 |
| 5 - 9 yrs | 2,933 (87.9) | 0.19 (0.08 – 0.47) | <0.001 |
| 10 - 14 yrs | 2,508 (85.4) | 0.13 (0.05 – 0.35) | <0.001 |
| 15 - 44 yrs | 6,796 (81.6) | 0.10 (0.04 – 0.26) | <0.001 |
| ≥ 45 yrs | 1,957 (82.6) | 0.12 (0.05 – 0.32) | <0.001 |

^a^Adjusted for age, gender, and cluster-level clustering

Note: Contact duration was categorised as lasting < 1 hour or ≥ 1 hour

# **ST D2: Association of age groups of participants and contactees with contact location**

| Age Category | Number of household contacts  n (%) | Adjusted Odds ratio^a^  (95% C1) | p-value |
| --- | --- | --- | --- |
| *Participant age group in months (m) or years (yrs)* |  |  |  |
| 0-11 m | 2,731 (89.4) | 1 (ref) |  |
| 12-23 m | 2,715 (89.8) | 1.04 (0.79 – 1.37) | 0.77 |
| 24-59 m | 2,657 (83.6) | 0.59 (0.46 – 0.77) | <0.001 |
| 5-9 yrs | 1,385 (73.2) | 0.32 (0.24 – 0.42) | <0.001 |
| 10-14 yrs | 1,238 (73.4) | 0.31 (0.24 – 0.42) | <0.001 |
| 15-44 yrs | 2,704 (78.4) | 0.40 (0.31 – 0.51) | <0.001 |
| ≥ 45 yrs | 2,695 (76.4) | 0.37 (0.29 – 0.48) | <0.001 |
| Age group of contacts in months (m) or years (yrs) |  |  |  |
| 0-11 m | 344 (96.4) | 1 (ref) |  |
| 12-23 m | 733 (93.4) | 0.79 (0.29 – 2.18) | 0.64 |
| 24-59 m | 1,339 (84.8) | 0.25 (0.10 – 0.62) | <0.01 |
| 5 - 9 yrs | 2,797 (83.7) | 0.15 (0.06 – 0.37) | <0.001 |
| 10 - 14 yrs | 2,360 (80.2) | 0.09 (0.04 – 0.23) | <0.001 |
| 15 - 44 yrs | 6,684 (80.1) | 0.10 (0.04 – 0.25) | <0.001 |
| ≥ 45 yrs | 1,841 (77.5) | 0.16 (0.05 – 0.34) | <0.001 |

^a^Adjusted for age, gender, and cluster-level clustering

Note: Contact location was categorised as household or non-household.

# **ST D3: Association of age groups of participants and contactees with the general regularity of contact**

| Age Category | Number of contacts that occur daily/almost daily  n (%) | Adjusted Odds ratio^a^  (95% C1) | p-value |
| --- | --- | --- | --- |
| *Participant age group in months (m) or years (yrs)* |  |  |  |
| 0-11 m | 2,669 (87.6) | 1 (ref) |  |
| 12-23 m | 2,713 (89.9) | 1.28 (0.97 – 1.67) | 0.07 |
| 24-59 m | 2,820 (89.0) | 1.13 (0.88 – 1.45) | 0.35 |
| 5-9 yrs | 1,636 (86.9) | 0.93 (0.68 – 1.26) | 0.62 |
| 10-14 yrs | 1,481 (87.4) | 0.99 (0.70 – 1.40) | 0.96 |
| 15-44 yrs | 2,854 (83.3) | 0.61 (0.47 – 0.81) | <0.01 |
| ≥ 45 yrs | 2,749 (78.1) | 0.48 (0.37 – 0.63) | <0.001 |
| Age group of contacts in months (m) or years (yrs) |  |  |  |
| 0-11 m | 353 (98.9) | 1 (ref) |  |
| 12-23 m | 763 (97.5) | 0.52 (0.08 – 3.34) | 0.49 |
| 24-59 m | 1,466 (93.0) | 0.11 (0.02 – 0.62) | 0.01 |
| 5 - 9 yrs | 3,066 (91.7) | 0.08 (0.02 – 0.40) | <0.01 |
| 10 - 14 yrs | 2,567 (87.3) | 0.04 (0.01 – 0.23) | <0.001 |
| 15 - 44 yrs | 6,678 (80.1) | 0.02 (0.00 – 0.14) | <0.001 |
| ≥ 45 yrs | 1,994 (84.0) | 0.04 (0.01 – 0.19) | <0.001 |

^a^Adjusted for age, gender, and cluster-level clustering

Note: Contact generality was categorised as infrequent or daily/almost daily

# **ST D4: Association of contact parameters with overall pneumococcal nasopharyngeal carriage**

| Contact characteristics | Number of contacts  n (%) | Crude Odds ratio  (95% C1) | | p-value | Adjusted Odds ratio^a^  (95% C1) | p-value |
| --- | --- | --- | --- | --- | --- | --- |
| *Frequency of contacts* |  |  | |  |  |  |
| Total number of contacts per 24 hours | 19,811 (100) | 0.97 (0.96 - 0.99) | | <0.01 | 0.99 (0.98 – 1.01) | 0.23 |
| *Contact type* |  |  | |  |  |  |
| Non-physical | 2,904 (14.6) | 1 (ref) | |  | 1 (ref) |  |
| Physical contact | 16,908 (85.4) | 1.13 (1.04 – 1.23) | | <0.01 | 1.03 (0.96 – 1.11) | 0.42 |
| *Location* |  |  | |  |  |  |
| Non-household | 3,686 (18.6) | 1 (ref) | |  | 1 (ref) |  |
| Household | 16,125 (81.4) | 1.24 (1.14 - 1.35) | | <0.01 | 1.11 (1.03 – 1.19) | <0.01 |
| *General Regularity* |  |  | |  |  |  |
| Infrequent | 2,823 (14.3) | 1 (ref) | |  | 1 (ref) |  |
| Daily/almost daily | 16,922 (85.7) | 1.14 (1.04 – 1.26) | | <0.01 | 1.02 (0.95 – 1.10) | 0.55 |
| *Duration* |  |  | |  |  |  |
| Contact < 1 hour | 2,968 (15.0) | 1 (ref) | |  | 1 (ref) |  |
| Contact ≥ 1 hour | 16,763 (85.0) | 1.17 (1.04 - 1.31) | | <0.01 | 1.01 (0.92 – 1.10) | 0.85 |
| *Number of physical contacts per 24 hours made with those:* |  | |  |  |  |  |
| < 2 years | 1,052 (6.2) | 0.94 (0.82 – 1.07) | | 0.36 | 0.93 (0.84 – 1.03) | 0.18 |
| 2 - 4 years | 1,490 (8.8) | 1.06 (1.01 – 1.12) | | 0.02 | 0.98 (0.94 – 1.02) | 0.31 |
| 5 - 9 years | 3,155 (18.7) | 1.04 (1.01 – 1.08) | | 0.02 | 1.00 (0.97 – 1.03) | 0.93 |
| 10 - 14 years | 2,564 (15.2) | 0.98 (0.93 – 1.03) | | 0.36 | 0.98 (0.94 – 1.01) | 0.22 |
| 15 - 44 years | 6,675 (39.5) | 0.96 (0.94 – 0.98) | | 0.01 | 1.00 (0.98 – 1.02) | 0.73 |
| 45+ years | 1,951 (11.6) | 0.90 (0.85 – 0.96) | | 0.01 | 1.00 (0.93 – 1.07) | 0.96 |
| ^#^0-4 years | 2,542 (15.0) | 1.02 (0.97 – 1.07) | | 0.45 | 0.98 (0.94 – 1.02) | 0.36 |
| ^#^5-14 years | 5,719 (33.9) | 1.01 (1.99 – 1.03) | | 0.22 | 0.99 (0.97 – 1.01) | 0.21 |

^a^Adjusted for age, gender, season, and antibiotic use

^#^Age-group of interest

# **ST D5: Unadjusted and adjusted odds ratios showing the association of risk factors with any pneumococci nasopharyngeal carriage in a cross-sectional carriage and contact survey conducted in 2022 (n = 1636)**

| Characteristics | | Crude Odds Ratio  (95% CI) | | P-value | Adjusted Odds Ratio  (95%CI) | p-value |
| --- | --- | --- | --- | --- | --- | --- |
| Age in months (m) or years (yrs) |  |  |  | |  |  |
|  | 0-11 m | 1 (ref) | |  | 1 (ref) |  |
|  | 12-23 m | 0.83 (0.59 – 1.17) | | 0.29 | 0.83 (0.59 – 1.18) | 0.29 |
|  | 24-59 m | 0.43 (0.31 – 0.60) | | <0.001 | 0.40 (0.28 – 0.57) | <0.001 |
|  | 5-9 yrs | 0.22 (0.15 – 0.32) | | <0.001 | 0.20 (0.14 – 0.31) | <0.001 |
|  | 10-14 yrs | 0.21 (0.14 – 0.32) | | <0.001 | 0.18 (0.11 – 0.28) | <0.001 |
|  | 15-44 yrs | 0.08 (0.05 – 0.13) | | <0.001 | 0.05 (0.03 – 0.08) | <0.001 |
|  | ≥ 45 yrs | 0.04 (0.03 – 0.07) | | <0.001 | 0.02 (0.01 – 0.04) | <0.001 |
| Sex |  |  | |  |  |  |
|  | Female | 1 (ref) | |  | 1 (ref) |  |
|  | Male | 1.46 (1.06 – 2.01) | | 0.02 | 1.46 (1.05 – 2.03) | 0.02 |
| Ethnicity |  |  | |  |  |  |
|  | Fula | 1.37 (0.46 – 4.08) | |  |  |  |
|  | Serahule | 2.47 (0.82 – 7.44) | |  |  |  |
|  | Mandinka | 2.05 (0.61 – 6.84) | | 0.61 |  |  |
|  | Wolof | 0.80 (0.23 – 2.78) | |  |  |  |
|  | Others | 1 (Reference) | |  |  |  |
| Season |  |  | |  |  |  |
|  | Rainy | 1 (ref) | |  | 1 (ref) |  |
|  | Dry | 2.41 (1.52 – 3.80) | | <0.001 | 2.27 (1.44 – 3.58) | <0.01 |
| ^e^compound size |  |  | |  |  |  |
|  | 0 - 19 | 1 (ref) | |  |  |  |
|  | 20 - 49 | 1.32 (0.89 – 1.97) | | 0.31 |  |  |
|  | ≥ 50 | 1.21 (0.76 – 1.94) | |  |  |  |
| Bed-sharing with a child aged < 10 yrs |  |  | |  |  |  |
|  | No | 1 (ref) | |  |  |  |
|  | Yes | 1.22 (0.93 – 1.60) | | 0.15 |  |  |
| Cooking location |  |  | |  |  |  |
|  | Inside | 1 (ref) | |  |  |  |
|  | Outside | 0.66 (0.26 – 1.65) | |  |  |  |
|  | Inside & outside | 1.21 (0.84 – 1.73) | | 0.32 |  |  |
| Smoker in the household |  |  | |  |  |  |
|  | No | 1 (ref) | |  |  |  |
|  | Yes | 1.10 (0.78 – 1.55) | | 0.57 |  |  |
| Has had a runny nose within the past two weeks |  |  | |  |  |  |
|  | No | 1 (ref) | |  | 1 (ref) |  |
|  | Yes | 1.35 (0.94 – 1.93) | | 0.10 | 1.42 (0.99 – 2.06) | 0.06 |
| Took antibiotics in the last two weeks |  |  | |  |  |  |
|  | No | 1 (ref) | |  | 1 (ref) |  |
|  | Yes | 0.63 (0.40 – 0.98) | | 0.04 | 0.59 (0.39 – 0.93) | 0.02 |

95% CI – 95% Confidence Interval adjusted for cluster-level clustering

Missing values: cooking location=7, cooking method=9, Has had a runny nose within the past two weeks=7

^a^ Covariates adjusted for were participant age group and gender

^e^ A compound consists of groups of families typically residing together in a living arrangement

# **ST D6: The contribution of different age groups toward age-specific exposure to vaccine-type pneumococcus**

| **Age group of contacts (yrs)** | **Participant age group (yrs)** | | | | | |
| --- | --- | --- | --- | --- | --- | --- |
|  | **< 2 years** | **2-4 yrs** | **5-9 yrs** | **10-14 yrs** | **15-44 yrs** | **≥ 45 yrs** |
| **< 2 yrs** | 3.5% (1.9 - 6.2) | 4.3% (3.0 - 6.3) | 3.9% (2.6 - 5.7) | 2.7% (1.5 - 4.7) | 5.9% (3.9 - 8.9) | 8.3% (5.6 - 11.9) |
| **2-4 yrs** | 17.2% (11.3 - 24.5) | 43.7% (28.7 - 57.5) | 11.2% (6.8 - 17.2) | 6.7% (2.9 - 13.1) | 9.8% (6.1 - 14.8) | 12.6% (8.2 - 18.3) |
| **5-9 yrs** | 48.5% (35.4 - 60.3) | 35.4% (23.5 - 48.9) | 65.1% (51.7 - 75.7) | 29.6% (17.8 - 44.9) | 24.9% (15.1 - 37.7) | 31.7% (20.9 - 43.4) |
| **10-14 yrs** | 17.5% (8.6 - 29.4) | 10.6% (4.5 - 21.3) | 15.1% (7.7 - 25.3) | 51.8% (33.0 - 67.8) | 25.8% (14.0 - 39.6) | 19.6% (10.5 - 30.4) |
| **15-44 yrs** | 9.7% (3.3 - 18.2) | 4.1% (1.3 - 7.9) | 3.4% (1.0 - 7.2) | 7.1% (2.3 - 15.1) | 29.3% (11.2 - 46.9) | 16.6% (5.9 - 29.1) |
| **≥ 45 yrs** | 2.6% (0.7 - 5.6) | 1.0% (0.3 - 2.1) | 0.8% (0.2 - 1.7) | 1.0% (0.3 - 2.2) | 3.3% (1.0 - 6.8) | 9.9% (3.0 - 19.8) |

Note: values denote mean and bootstrapped 95% confidence intervals over 10,000 bootstrap samples of the estimated proportion of all contacts made by a participant of age j (columns) with contacts carrying pneumococci of age i (rows).

# **ST D7: Unadjusted and adjusted odds ratios showing the association of risk factors with PCV13 vaccine-type pneumococcal nasopharyngeal carriage in a cross-sectional carriage and contact survey conducted in 2022 (n = 1636)**

| Characteristic5 | | Crude Odds Ratio  (95% CI) | | P-value | Adjusted Odds Ratio  (95%CI) | p-value |
| --- | --- | --- | --- | --- | --- | --- |
| Age in months (m) or years (yrs) |  |  |  | |  |  |
|  | 0-11 m | 1 (ref) | |  | 1 (ref) |  |
|  | 12-23 m | 0.87 (0.51 – 1.48) | | 0.60 | 0.83 (0.47 – 1.45) | 0.51 |
|  | 24-59 m | 0.97 (0.60 – 1.56) | | 0.89 | 0.85 (0.51 – 1.45) | 0.53 |
|  | 5-9 yrs | 1.43 (0.86 – 2.37) | | 0.17 | 1.24 (0.74 – 2.09) | 0.40 |
|  | 10-14 yrs | 1.00 (0.51 – 1.97) | | 0.99 | 0.77 (0.38 – 1.56) | 0.46 |
|  | 15-44 yrs | 0.22 (0.08 – 0.58) | | <0.01 | 0.12 (0.05 – 0.33) | <0.001 |
|  | ≥ 45 yrs | 0.19 (0.08 – 0.47) | | <0.001 | 0.10 (0.04 – 0.27) | <0.001 |
| Sex |  |  | |  |  |  |
|  | Female | 1 (ref) | |  | 1 (ref) |  |
|  | Male | 1.75 (1.01 – 3.04) | | 0.04 | 1.75 (1.01 – 3.05) | 0.0.4 |
| Ethnicity |  |  | |  |  |  |
|  | Fula | 4.71 (0.41 – 53.62) | |  |  |  |
|  | Serahule | 7.98 (0.71 – 89.16) | |  |  |  |
|  | Mandinka | 7.27 (0.61 – 86.65) | | 0.53 |  |  |
|  | Wolof | 4.33 (0.34 – 54.54) | |  |  |  |
|  | Others | 1 (ref) | |  |  |  |
| Season |  |  | |  |  |  |
|  | Rainy | 1 (ref) | |  | 1 (ref) |  |
|  | Dry | 2.07 (1.12 – 3.82) | | 0.02 | 1.99 (1.07 – 3.69) | 0.03 |
| ^e^Compound size |  |  | |  |  |  |
|  | 0 - 19 | 1 (ref) | |  |  |  |
|  | 20 - 49 | 0.90 (0.50 – 1.61) | | 0.55 |  |  |
|  | ≥ 50 | 0.76 (0.30 – 1.92) | |  |  |  |
| Bed-sharing with a child aged < 10 yrs |  |  | |  |  |  |
|  | No | 1 (ref) | |  |  |  |
|  | Yes | 1.51 (0.86 – 2.66) | | 0.15 |  |  |
| Cooking location |  |  | |  |  |  |
|  | Inside | 1 (ref) | |  |  |  |
|  | Outside | 0.98 (0.17 – 5.61) | |  |  |  |
|  | Inside & outside | 1.32 (0.77 – 2.28) | | 0.32 |  |  |
| Smoker in the household |  |  | |  |  |  |
|  | No | 1 (ref) | |  |  |  |
|  | Yes | 0.94 (0.55 – 1.59) | | 0.81 |  |  |
| Has had a runny nose within the past two weeks |  |  | |  |  |  |
|  | No | 1 (ref) | |  |  |  |
|  | Yes | 1.12 (0.61 – 2.03) | | 0.71 |  |  |
| Took antibiotics in the last two weeks |  |  | |  |  |  |
|  | No | 1 (ref) | |  | 1 (ref) |  |
|  | Yes | 0.47 (0.27 – 0.83) | | <0.01 | 0.51 (0.29 – 0.89) | 0.02 |

95% CI – 95% Confidence Interval adjusted for cluster-level clustering

Missing values: cooking location=7, cooking method=9, Has had a runny nose within the past two weeks=7

^a^ Covariates adjusted for were participant age group and gender

^e^ A compound consists of groups of families typically residing together in a living arrangement
